# Effect assessment of the application value of evidence-based nursing intervention in operating room nursing: A protocol for a systematic review and meta-analysis

**DOI:** 10.1097/MD.0000000000026867

**Published:** 2021-08-13

**Authors:** You Zhou, Xin Li

**Affiliations:** Operation Room, Wuhan Fourth Hospital, Puai Hospital, Tongji Medical College, Huazhong University of Science and Technology, Wuhan, Hubei, China.

**Keywords:** application, evidence-based, meta, nursing, operating room

## Abstract

**Background:**

The advantages of evidence-based nursing (EBN) intervention in health care settings have been widely disseminated to nurses throughout the world. More researches are reporting the effectiveness of EBN intervention in operating room nursing. However, the results are inconsistent. This study focuses on conducting a meta-analysis and systematic evaluation aimed at determining the usefulness of EBN intervention in operating room nursing.

**Methods:**

The Preferred Reporting Items for Systematic Reviews and Meta-analysis for Protocols criteria were used to write this paper. We will look for relevant studies from 2 Chinese databases (China National Knowledge Infrastructure and Wanfang database and also from 3 English databases such as Web of Science, Cochrane Library, PubMed, and EMBASE), to locate all relevant randomized controlled trials and observational studies assessing the application value of EBN intervention in operating room nursing from their commencement to June 2021. Separately, 2 authors will choose the studies, do the data extract and conduct the assessment probing into the likelihood of bias. If there is a disagreement, it will be resolved by the third author. RevMan 5.3 software and Stata 15.0 software will be used to conduct the meta-analysis.

**Results:**

The usefulness of EBN intervention in operating room nursing will be assessed in this study.

**Conclusion:**

The purpose of this research is to conclude the value of EBN intervention in operating room nursing and the quality of current data.

**Ethics and dissemination:**

Since there is no requirement for data on the individual patient, hence there will be no need for ethical approval.

**OSF Number:**

DOI 10.17605/OSF.IO/MSXNF

## 1 Introduction

The need for highly educated nurses has increased dramatically due to progress in science and technology, as well as the evolution of nursing models, and the number of operating room nurses and other medical care services has also increased.^[1]^ As a result, optimizing the training process and fostering the best professional quality of operating room nurses as rapidly as possible has become crucial in nursing education. Furthermore, the intensive characteristics of operating rooms, such as emergencies, strict sterility standards, and strong specialization, create a particularly high demand for operating room nurses’ workload.^[2]^ During surgery, the technical performance of operating room professionals, such as anesthesiologists, surgeons, and nurses, can have a direct impact on patient safety and health outcomes.^[3,4]^ The operating room nurse's duty for patient safety has been extended and now includes the time before and after the operation.^[5]^ Operating room nursing involves high load, high risk, an enormous responsibility, and high pressure due to these factors.

Nurses in operating rooms not only have to deal with a lot of workloads but they also have to deal with a lot of occupational hazards, which may quickly lead to physical and mental exhaustion.^[6]^ In recent research, operating room nurses were shown to have more job-related pressures and greater levels of depression and anxiety than ward nurses.^[7]^ As a result, research on this topic has significant clinical implications. Evidence-based nursing (EBN) is currently the global standard. Despite the fact that multiple studies demonstrate that nurses are aware of the necessity and advantages of EBN intervention for healthcare systems, patients, and themselves, integrating EBN intervention in nursing practice has been a slow process.^[8–10]^ The usefulness of EBN intervention in operating room nursing, on the other hand, remains unclear. As a result, we will perform a direct and thorough systematic evaluation to determine the usefulness of EBN intervention in operating room nursing.

## 2 Methods

The registration of this protocol can be found on OSF, http://osf.io/). The PRISMA-P statement guidelines will guide our protocol.

### 2.1 Study selection criteria (inclusion criteria)

#### 2.1.1 Different kinds of studies

All randomized controlled trials (RCTs) and observational studies examining the efficacy of EBN intervention in operating room nursing will be considered. Reviews, animal experiments, laboratory studies, and case reports will not be included.

#### 2.1.2 Kinds of participants

This research will only involve those who have had surgery under general anesthesia or spinal anesthesia. There will be no limits based on age at onset, gender, ethnicity, or educational background.

#### 2.1.3 Types of interventions

EBN intervention will be compared against no nursing intervention or other kinds of nursing intervention in trials.

#### 2.1.4 Types of outcome measures

Patient satisfaction, complication rate, quality of treatment in the operating room, and adverse events are the study's objectives.

### 2.2 Identification of studies and the search methods

#### 2.2.1 Searches based on an electronic approach

We will look for all relevant RCTs and observational studies evaluating the application value of EBN intervention in operating room nursing in 3 English databases (PubMed, EMBASE, the Cochrane Library, and Web of Science) and 2 Chinese databases (Wanfang database and China National Knowledge Infrastructure) from their inception to June 2021. The related terms are as follows: “evidence-based,” “operating room,” and “nursing.” Languages were restricted to Chinese and English.

#### 2.2.2 Searching other resources

We will also look through Google Scholar and the gray literature to see if there are any research on EBN intervention that are relevant. For related journals and conference proceedings, manual searches will be done.

### 2.3 Data collection and analysis

#### 2.3.1 Selection of studies

**Figure 1:**
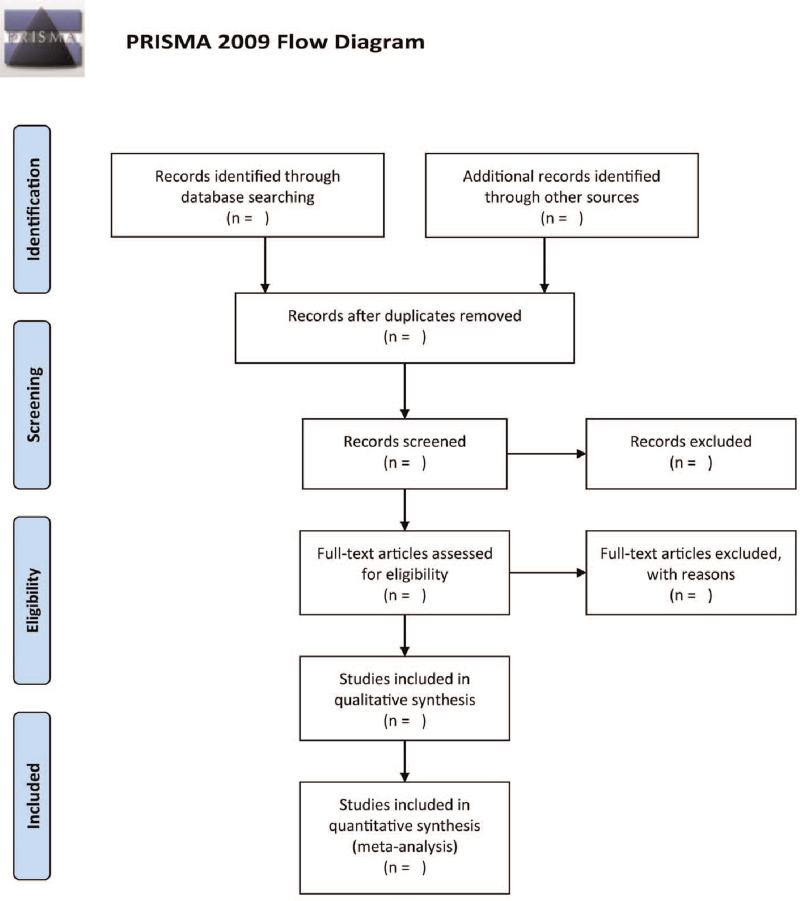
Flow diagram of literature for systematic review and meta-analysis.

The studies will be screened and selected by 2 independent authors. The reviews will first screen the titles and abstracts, then exclude irrelevant papers. Second, we will get all of the remaining studies’ full-text articles. Following that, the authors will examine the studies to determine if they are eligible for inclusion and to describe the reasons for study exclusion. If there are any differences, they will be handled by consensus or by involving a third author. The study's selection approach is depicted in Figure 1.

#### 2.3.2 The management of data and its extraction

The data will be extracted using a standardized form based on the inclusion criteria by 2 autonomous scholars. Duplicate items will be eliminated from the EndNote X9 software when the search results are entered. Study characteristics (study type, first author, publication date, patient characteristics, sample size and number of patients (mean age, gender, and medical history), intervention features, and outcome measures will be included in the extract data.

#### 2.3.3 Bias risk and evaluation

Using the Cochrane Risk of Bias Tool, 2 researchers will quantify the risk of bias independently.^[11]^ The methodological quality of the observational studies included will be assessed using the Newcastle–Ottawa Scale.^[12]^

#### 2.3.4 Effect of treatment measures

For dichotomous data, we will compute risk ratios and 95% confidence intervals (CIs). To examine continuous outcomes data, we will use a 95% weighted mean difference or standardized mean difference.

#### 2.3.5 Assessment of heterogeneity

The *I*^2^ statistic will be used to measure heterogeneity before any outcome is aggregated. We will utilize the random-effects model for analysis if there is sufficient heterogeneity (*I*^2^ > 50%); otherwise, we will use the fixed-effect model.

#### 2.3.6 Assessment of reporting bias

We will do a full protocol assessment to look at reporting biases. Because the number of included studies may reach 10, the funnel plot is ideal for analyzing publication bias.

#### 2.3.7 Sensitivity analysis

The effect of exclusion for the research with a risk bias of high value will be assessed using a sensitivity analysis.

## 3 Discussion

This will be the first systematic review to synthesize the research on the usefulness of EBN intervention in operating room nursing. More information concerning the value of EBN intervention in operating room nursing will be provided. Following data synthesis, the findings of this assessment will give a clear basis and direction for ongoing study in the areas that are considered to require greater examination. To guarantee the complete openness of the process, this protocol gives a clear overview of the reasoning and methodology to be employed in the proposed systematic review. This study presents no ethical concerns, and any potential biases in the review process will be disclosed in the final review paper's discussion section. A comprehensive search approach of published literature will be used as one of the protocol's strengths. Each analysis’ total findings will be reviewed qualitatively and quantitatively. To completely assess the application value of EBN intervention in operating room nursing and strengthen the article content's reliability and findings, the causes of heterogeneity and distinct subgroups of the articles will be evaluated. We anticipate that patients undergoing surgery, physicians, healthcare administrators, and policymakers would benefit from this systematic review.

## Author contributions

**Conceptualization:** You Zhou, Xin Li.

**Data curation:** You Zhou, Xin Li.

**Formal analysis:** You Zhou, Xin Li.

**Funding acquisition:** You Zhou, Xin Li.

**Methodology:** You Zhou, Xin Li.

**Resources:** You Zhou, Xin Li.

**Software:** Xin Li.

**Supervision:** You Zhou.

**Validation:** Xin Li.

**Visualization:** You Zhou, Xin Li.

**Writing – original draft:** Xin Li.

**Writing – review & editing:** You Zhou, Xin Li.
